# Predictors of Appropriate Antibiotic Use in Bacteremia Patients Presenting at the Emergency Department

**DOI:** 10.3390/antibiotics8030142

**Published:** 2019-09-09

**Authors:** Pariwat Phungoen, Areerat Kraisriwattana, Korakot Apiratwarakul, Lumyai Wonglakorn, Kittisak Sawanyawisuth

**Affiliations:** 1 Department of Emergency Medicine, Faculty of Medicine, Khon-Kaen University, Khon Kaen 40002, Thailand (P.P.) (A.K.) (K.A.); 2 Sleep Apnea Research Group, Research Center in Back, Neck and Other Joint Pain and Human Performance, Research and Training Center for Enhancing Quality of Life of Working Age People, and Research and Diagnostic Center for Emerging Infectious Diseases (RCEID), Khon Kaen University, Khon Kaen 40002, Thailand; 3 Clinical Microbiology Laboratory, Srinagarind Hospital, Faculty of Medicine, Khon Kaen University, Khon Kaen 40002, Thailand; 4 Department of Medicine, Faculty of Medicine, Khon-Kaen University, Khon Kaen 40002, Thailand

**Keywords:** sepsis, pulse rate, predictors, mortality, hospitalization, community-acquired infection

## Abstract

Sepsis is a condition that requires appropriate antibiotic treatment at the emergency department (ED). Most previous studies conducted on inappropriate antibiotic use at the ED were conducted in developed countries with a low percentage of sepsis. This study aimed to find additional clinical predictors for appropriate antibiotic use in bacteremia patients presenting at the ED from a developing country, in which there is a higher proportion of patients with sepsis. We included adult patients who presented at the ED with clinical suspicion of infection and bacteremia. Patients allocated to the appropriate antibiotic group were those in whom the prescribed antibiotic was sensitive to the pathogen. Predictors and outcomes of appropriate antibiotic use were analyzed. A total of 3133 patients who met the study criteria presented at the ED during the study period. Of those, 271 patients were diagnosed with bacteremia, 48 of whom (17.71%) received inappropriate antibiotic prescriptions. Only pulse rate was an independent factor for appropriate antibiotic treatment, with an adjusted odds ratio of 1.019 (95% CI of 1.001, 1.036). In terms of clinical outcomes, the inappropriate antibiotic group had higher proportions of 28-day mortality (29.17% vs. 25.25%; *p*-value = 0.022) and longer hospitalization (14 vs. 9 days; *p*-value = 0.003). This study found that inappropriate antibiotics were prescribed in 17% of bacteremia patients presenting at the ED and that high pulse rate was an indicator for appropriate antibiotic prescription. Patients with inappropriate antibiotic administration had longer hospitalization and higher 28-day mortality than those who received appropriate antibiotic treatment.

## 1. Introduction

Sepsis is a life-threatening condition that is a major problem worldwide. In the United States, there were over 750,000 cases in 1995, 61% of whom were admitted through the emergency department (ED) [1,2]. In China, the number of sepsis patients was even greater at 1,256,684 between 2001 and 2012, 29.3% of whom were admitted via the ED [3]. With so many sepsis patients presenting at the ED, prompt and appropriate management in this department is crucial.

Appropriate and adequate antibiotic treatment is one of the key factors that determine the outcomes in cases of sepsis [4]. Although inappropriate antibiotic treatment has been shown to increase the mortality rate by five times in these patients [5], the rate of inappropriate antibiotic administration at the ED may be as high as 40% [4,5,6,7]. A previous study found that patients with co-morbid diseases, such as COPD or hemodialysis, or who were immunocompromised were at risk of receiving inappropriate antibiotic treatment at the ED [8]. Most previous studies conducted on inappropriate antibiotic use at the ED were conducted in developed countries [4,5,6,7]. Additionally, a recent study included only the 4.7% of patients (32/678) who met the criteria specified in the quick Sepsis-Related Organ Failure Assessment (qSOFA) [7]. This study aims to highlight additional clinical predictors for appropriate antibiotic use in bacteremia patients presenting at the emergency department from a developing country, in which there is a higher proportion of patients with sepsis. 

## 2. Methods

This was a descriptive, analytical, retrospective study that was conducted at the ED of Khon Kaen University’s university hospital (Thailand). The inclusion criteria were: Age over 18 years, having presented at the ED with clinical suspicion of infection as diagnosed by the emergency physician, having provided at least two sets of blood cultures, and infection with a bacterial pathogen. Patients with a history of antibiotic administration or with contaminated/non-bacterial pathogens were excluded. This study was a part of the SEPSIS project at Khon Kaen University’s ED. Patients were enrolled between January and July, 2018. The study protocol was approved by the ethics committee in human research, Khon Kaen University, Thailand (HE611518).

The pathogen was identified by positive blood culture with a similar pathogen for at least one sample with clinical relevance, while contaminated pathogens were those without clinical relevance. Eligible patients were divided into two groups: Those who were given appropriate antibiotic treatment and those whose antibiotic treatment was inappropriate. Patients allocated to the appropriate antibiotic group were those in whom the prescribed antibiotic was sensitive to the pathogen, based on an antimicrobial susceptibility test reported by the blood cultures. The charts and emergency medical records of eligible patients were reviewed retrospectively. Baseline clinical characteristics and clinical outcomes were evaluated. Outcomes included antibiotic treatment within 60 min, mortality rate, intensive care unit (ICU) admission, and length of hospital stay.

Sample size calculation: Based on a population proportion estimation with specified absolute precision, the acceptable incidence of appropriate antibiotic administration was 80%. We determined that the required sample size was 246 patients, with a confidence of 95% and a power of 80%.

Statistical analysis: Descriptive statistics were used to evaluate differences between the two groups. Factors associated with appropriate antibiotic administration were analyzed using univariate and multivariate logistic regression analysis. Clinically relevant factors or those with a *p*-value of less than 0.20, based on univariate logistic regression analysis, were included in subsequent multivariate logistic regression analysis. The final model was tested for goodness of fit using the Hosmer–Lemeshow method. Results were presented as adjusted and unadjusted odds ratios (95% confidence interval, CI) for appropriate antibiotic prescription. Statistical analyses were performed using STATA version 10.1 (College Station, TX, USA).

## 3. Results

A total of 3133 patients who met the study criteria presented at the ED during the study period. Of those, 2862 were excluded due to negative hemoculture (*n* = 2674), contaminated blood culture results (*n* = 80), or fungal infection (*n* = 2), leaving 271 to undergo analysis. The three most common sources of infection were urinary tract infection (20.30%), biliary tract infection (16.97%), and primary bacteremia (16.61%), as shown in Table 1. *Escherichia coli* was the most common pathogen (33.21%).

Forty-eight of the patients enrolled (17.71%) received inappropriate antibiotic prescriptions (Table 2). There were two significant factors that differed between patients who received appropriate antibiotic treatment and those who were given inappropriate antibiotics: History of hypertension and body temperature. The inappropriate antibiotic group had a higher proportion of hypertensive patients (45.83% vs. 29.60%; *p*-value = 0.029), and patients in the appropriate antibiotic group had significantly higher body temperature (38.31 vs. 37.80oC; *p*-value = 0.023). However, only pulse rate was an independent factor for appropriate antibiotic treatment after adjusting for other factors, as shown in Table 3. The adjusted odds ratio was 1.019 with a 95% CI of 1.001, 1.036. The Hosmer–Lemeshow Chi square for the final model was 11.97 (*p*-value = 0.157).

In terms of clinical outcomes, the inappropriate antibiotic group had higher proportions of 28-day mortality (29.17% vs. 25.25%; *p*-value = 0.022) than the appropriate antibiotic group and hospital admission of greater than or equal to seven days (77.08% vs. 54.26%; *p*-value = 0.004) (Table 4). The median duration of hospital admission was also significantly longer in the inappropriate antibiotic group (14 vs. 9 days; *p*-value = 0.003). 

## 4. Discussion

The prevalence of inappropriate antibiotic prescription at the ED was 17.71%, which was comparable with that found in another study conducted at Massachusetts General Hospital in the United States (18%) [5], but lower than those in two other reports from the United States (31.2%) and Australia (32.7%) [6,7]. These differences may have been due to factors such as study population and resistant organisms. One previous study, for example, showed that appropriate antibiotic use may be higher in children than in adults (77.1% vs. 22.9%) [7], and another found that 25 out of 137 patients had resistant organisms, leading to ineffective antibiotic treatment [5].

We found that inappropriate antibiotic administration increased the risk of mortality and length of hospital stay, which is consistent with the findings of the latter report (Table 4) [5]. In addition, one study from the United States found that patients given inappropriate antibiotics had a higher mortality rate than those administered appropriate antibiotics (20.8% vs. 20.6%) [6]. We also found that pulse rate was the most important predictor of appropriate antibiotic administration. However, unlike history of hypertension or body temperature, this was not statistically significant according to univariate logistic analysis. This may imply that pulse rate was a stronger predictor of appropriate antibiotic use than the other two factors when adjusted for other factors. Pulse rate has been known for early sepsis resulting in more aggressive or even early antibiotic use in sepsis patients at the ED. A study that employed time-series monitoring at the intensive care unit found that a rise in heart rate was a predictor of sepsis four hours before clinical detection, and that the sensitivity of the heart rate model was 85% combined with other clinical factors [9]. Additionally, a previous study found heart rate variability (HRV) to be an indicator for mortality in sepsis patients [10], being significantly higher in patients who died than in those who survived (31.8 s vs. 23.7 s; *p*-value = 0.02). In this study, for each 10-beat increase in pulse rate, the risk of inappropriate antibiotic administration went up by 19% (Table 3).

This study also found that the inappropriate antibiotic treatment may increase the duration of hospital admission (14 days) and the 28-day mortality rate (29.17%). Although delayed antibiotic administration can result in poor outcomes [11], these results were not due to this factor (p 0.134), as shown in Table 4. A previous systematic review also showed that inappropriate antibiotic treatment in sepsis patients increased the risk of 30-day mortality, with an adjusted odds ratio of 1.60 and 95% CI of 1.37 to 1.86. Our findings were similar in that inappropriate antibiotic therapy was only related to short-term (28- to 30-day) mortality (Table 4) and hospitalization. Long-term mortality may have been influenced by other factors, resulting in the non-significant findings.

The main strength of this study is that most of the patients enrolled (189/271) were likely to have sepsis. Approximately 70% of patients had a qSOFA score that met the criteria of sepsis, as shown in Table 2. There were some limitations to this study. First, some data may be missing due to the retrospective study design. In addition, the antibiotic judgments were made clinically by the attending physicians. There is currently no guideline for antibiotic use in our hospital. Finally, these results represented overall outcomes from a referral university hospital in Thailand and, thus, may only be applicable to settings in other developing countries.

## 5. Conclusions

Inappropriate antibiotics were prescribed in 17% of bacteremia patients presenting at the ED. High pulse rate was an indicator for appropriate antibiotic prescription. Patients presenting at the ED with clinical suspicion of infection and positive blood culture who underwent inappropriate antibiotic administration had longer hospitalization and higher 28-day mortality than those who received appropriate antibiotic treatment.

## Figures and Tables

**Table 1:** Sources of infection and organisms in patients presenting with bacteremia at the emergency department (*n* = 271).

Factors	No. (%)
Source of infection, no. (%)	
Urinary tract infection	55 (20.30%)
Biliary tract infection	46 (16.97%)
Primary bacteremia	45 (16.61%)
Chest infection (Pneumonia, bronchiectasis)	41 (15.13%)
Skin or soft-tissue infection	36 (13.28%)
Intra-abdominal infection	24 (8.86%)
Catheter-related infections	3 (1.11%)
Other (CNS infection, arthritis, endocarditis)	21 (7.75%)
Organisms	
Gram-negative bacteria, no. (%)	
*Escherichia coli*	90 (33.21%)
*Klebsiella*	39 (14.39%)
*Enterobacter*	14 (5.18%)
*Burkholderia pseudomallei*	8 (2.95%)
*Pseudomonas*	6 (2.21%)
*Acinetobacter baumannii*	6 (2.21%)
*Proteus mirabilis*	3 (1.11%)
*Citrobacter*	2 (0.74%)
Gram-positive bacteria, no. (%)	
*Streptococcus*	25 (9.25%)
Methicillin-sensitive *Staphylococcus aureus*	17 (6.27%)
Methicillin-resistant *Staphylococcus aureus*	3 (1.11%)

**Table 2:** Baseline characteristics of patients presenting with bacteremia at the emergency department, categorized by appropriateness of antibiotic prescription.

Factors	Appropriate Antibiotics *n* = 223	Inappropriate Antibiotics *n* = 48	*p*-Value
Age, years	60.85 (15.54)	63.02 (16.14)	0.259
Male sex, n (%)	123 (55.16)	20 (41.67)	0.089
Charlson comorbidity score	4.26 (2.75)	4.88 (2.99)	0.137
Comorbidity, n (%)			
Diabetes	76 (34.08)	16 (33.33)	0.921
Cancer	71 (31.84)	19 (39.54)	0.301
Hypertension	66 (29.60)	22 (45.83)	0.029
Cirrhosis	50 (22.42)	5 (10.42)	0.061
CKD	27 (12.11)	6 (12.50)	0.940
Heart failure	18 (8.07)	4 (8.33)	0.999
On immunosuppressive therapy	8 (3.59)	3 (6.25)	0.418
Connective tissue disease	7 (3.14)	3 (6.25)	0.389
Chronic lung disease	6 (2.69)	1 (2.08)	0.999
AIDS	3 (1.35)	1 (2.08)	0.544
Others	22 (9.87)	4 (8.33)	0.999
Type of infection			
Community, n (%)	220 (98.65)	48 (100)	0.999
Body temperature, °C	38.31 (22.72)	37.80 (1.30)	0.023
Pulse rate, bpm	107.05 (22.72)	98.22 (26.92)	0.096
Respiratory rate, tpm	26.45 (7.22)	27.60 (12.16)	0.814
SBP, mmHg	120.34 (29.96)	116.52 (36.78)	0.291
DBP, mmHg	70.26 (18.32)	68.25 (19.30)	0.241
MAP, mmHg	86.95 (21.08)	84.37 (23.30)	0.247
Shock, n (%)	63 (28.25)	11 (22.92)	0.697
Glascow coma scale	14.30 (2.08)	14.45 (1.35)	0.699
Saturation, %	93.98 (9.54)	95.64 (4.17)	0.376
WBC, /mm^3^	13897 (9411)	15610 (11401)	0.504
Band, %	2.15 (5.51)	1.31 (2.90)	0.858
Lactate, mg/dL	3.79 (3.19)	2.89 (1.97)	0.101
qSOFA, n (%)	154 (69.06)	35 (72.92)	0.121

Note. Data presented as mean (SD), unless indicated otherwise; CKD: Chronic kidney disease; SBP: Systolic blood pressure; DBP: Diastolic blood pressure; MAP: Mean arterial pressure; WBC: White blood cell; qSOFA: Quick Sepsis-Related Organ Failure Assessment.

**Table 3:** Predictors of appropriate use of antibiotics in patients presenting with bacteremia at the emergency department.

Factors	Unadjusted Odds Ratio (95% Confidence Interval)	Adjusted Odds Ratio (95% Confidence Interval)
Age	0.990 (0.970, 1.011)	1.007 (0.982, 1.032)
Male sex	1.722 (0.915, 3.238)	1.491 (0.704, 3.159)
Hypertension	0.496 (0.262, 0.938)	0.621 (0.262, 1.469)
Cirrhosis	2.485 (0.934, 6.609)	2.581 (0.798, 8.344)
Diabetes	1.034 (0.533, 2.002)	1.240 (0.537, 2.778)
Cancer	0.712 (0.374, 1.356)	0.878 (0.392, 1.967)
Body temperature	1.374 (1.066, 1.770)	1.240 (0.909, 1.692)
Pulse rate	1.016 (1.002, 1.029)	1.019 (1.001, 1.036)
Lactate	1.143 (0.975, 1.339)	1.092 (0.914, 1.304)

**Table 4:** Clinical outcomes of patients presenting with bacteremia at the emergency department who were given appropriate and inappropriate antibiotic treatment.

Outcomes	Appropriate Antibiotics *n* = 223	Inappropriate Antibiotics *n* = 48	*p*-Value
Received antibiotics within 60 min	137 (61.43%)	35 (72.92%)	0.134
28-day mortality	34 (15.25%)	14 (29.17%)	0.022
90-day mortality	50 (22.42%)	17 (35.42%)	0.058
ICU admission	87 (39.01%)	87 (39.01%)	0.255
Median hospital LOS, days (Min, Max)	9 (1, 81)	14 (2, 65)	0.003
Hospital LOS ≥ 7 days	121 (54.26)	37 (77.08)	0.004
Hospital LOS ≥ 21 days	25 (11.21%)	7 (14.58%)	0.511

Note. LOS: length of stay.

## References

[1] Singer M., Deutschman C.S., Seymour C.W., Shankar-Hari M., Annane D., Bauer M., Bellomo R., Bernard G.R., Chiche J.D., Coopersmith C.M. (2016). The Third International Consensus Definitions for Sepsis and Septic Shock (Sepsis-3). JAMA.

[2] Nguyen H.B., Rivers E.P., Abrahamian F.M., Moran G.J., Abraham E., Trzeciak S., Huang D.T., Osborn T., Stevens D., Talan D.A. (2006). Severe sepsis and septic Shock: Review of the literature and emergency department management guidelines. Ann. Emerg. Med..

[3] Yu C.W., Chang S.S., Lai C.C., Wu J.Y., Yen D.W., Lee M.G., Yeh C.C., Chung J.Y., Lin Y.J., Lee C.C. (2019). Epidemiology of Emergency Department Sepsis: A National Cohort Study Between 2001 and 2012. Shock.

[4] Rhodes A., Evans L.E., Alhazzani W., Levy M.M., Antonelli M., Ferrer R., Kumar A., Sevransky J.E., Sprung C.L., Nunnally M.E. (2017). Surviving Sepsis Campaign: International Guidelines for Management of Sepsis and Septic Shock: 2016. Intensive Care Med..

[5] Capp R., Chang Y., Brown D.F.M. (2011). Effective antibiotic treatment prescribed by emergency physicians in patients admitted to the intensive care unit with severe sepsis or septic Shock: Where is the gap?. J. Emerg. Med..

[6] Vilella A.L., Seifert C.F. (2014). Timing and appropriateness of initial antibiotic therapy in newly presenting septic patients. Am. J. Emerg. Med..

[7] Denny K.J., Gartside J.G., Alcorn K., Cross J.W., Maloney S., Keijzers G. (2019). Appropriateness of antibiotic prescribing in the Emergency Department. J. Antimicrob. Chemother..

[8] Allison M.G., Heil E.L., Hayes B.D. (2017). Appropriate Antibiotic Therapy. Emerg. Med. Clin. N. Am..

[9] Shashikumar S.P., Stanley M.D., Sadiq I., Li Q., Holder A., Clifford G.D., Nemati S. (2017). Early sepsis detection in critical care patients using multiscale blood pressure and heart ratedynamics. J. Electrocardiol..

[10] Prabhakar S.M., Tagami T., Liu N., Samsudin M.I., Ng J.C.J., Koh Z.X., Ong M.E.H. (2019). Combining quick sequential organ failure assessment score with heart rate variability may improve predictive ability for mortality in septic patients at the emergency department. PLoS ONE.

[11] Zasowski E.J., Claeys K.C., Lagnf A.M., Davis S.L., Rybak M.J. (2016). Time Is of the Essence: The Impact of Delayed Antibiotic Therapy on Patient Outcomes in Hospital-Onset Enterococcal Bloodstream Infections. Clin. Infect. Dis..

